# The first human pocket histology at an eight-week cardiac implantable electronic device with absorbable antibacterial envelope: case report

**DOI:** 10.1093/ehjcr/ytaf651

**Published:** 2025-12-18

**Authors:** Kee Koon Ng, Ching-Fen Chang, Yin-Huei Chen, Kuan-Cheng Chang, Yen-Nien Lin

**Affiliations:** Division of Cardiovascular Medicine, Department of Internal Medicine, China Medical University Hospital, Taichung 40447, Taiwan; Division of Cardiovascular Medicine, Department of Internal Medicine, China Medical University Hospital, Taichung 40447, Taiwan; Division of Endocrinology and Metabolism, Department of Internal Medicine, China Medical University Hospital, Taichung 40447, Taiwan; Division of Cardiovascular Medicine, Department of Internal Medicine, China Medical University Hospital, Taichung 40447, Taiwan; School of Medicine, China Medical University, Taichung 404333, Taiwan; Division of Cardiovascular Medicine, Department of Internal Medicine, China Medical University Hospital, Taichung 40447, Taiwan; School of Medicine, China Medical University, Taichung 404333, Taiwan

**Keywords:** Cardiac implantable electronic device, TYRX antibacterial envelope, Histopathology, Case report, Prophylaxis

## Abstract

**Background:**

The second-generation TYRX™ absorbable antibacterial envelope (Medtronic, Minneapolis, MN, USA) effectively reduces cardiac implantable electronic device (CIED) infections, as shown in the WRAP-IT trial. However, data on its degradation process and pocket histopathology in humans remain scarce, with most evidence derived from preclinical studies.

**Case summary:**

An 80-year-old woman with hypertension and hyperlipidaemia received a cardiac resynchronization therapy pacemaker for heart failure with reduced ejection fraction and left bundle branch block. The device was implanted with a TYRX envelope. Fifty-six days later, she presented with intermittent loss of left ventricular pacing due to atrial undersensing and underwent right atrial lead revision. Pocket exploration revealed a thin light amber capsule covering the device. Histopathology showed different stages of TYRX resorption and wound healing: residual envelope material with lymphocytes and multinucleated giant cells in the outer layer (chronic inflammation), collagen-rich granulation tissue with neovascularization in the middle layer (proliferation), and a dense acellular fibrous capsule adjacent to the device (remodelling). The maximum thickness of capsule measured 1210 μm.

**Discussion:**

This case provides rare human histopathological evidence of an eight-week-old TYRX envelope, illustrating ongoing degradation and distinct wound-healing phases. The findings bridge preclinical observations and human data, enhancing understanding of envelope absorption dynamics.

Learning points

- This case represents the first human histopathological observation of a cardiac implantable electronic device pocket eight weeks after implantation with a TYRX absorbable antibacterial envelope.
- The findings demonstrate incomplete envelope degradation with layered inflammatory and fibrotic responses, providing new insight into pocket healing and infection prevention mechanisms.

## Introduction

The second generation TYRX (Medtronic, Minneapolis, USA) has been used for reducing cardiac implantable electronic devices (CIED) infection for at least 5 years. TYRX is constructed from a multifilament knitted mesh coated with tyrosine-based polymer mixed with minocycline and rifampin.^1^ The antibiotics are locally released 2 h after implantation and last for more than 7 days while the envelope fully degraded in approximately 9 weeks.^1^ World-wide Randomized Antibiotic Envelope Infection Prevention trial (WRAP-IT) is a global, multicenter, prospective, single blind, randomized controlled trial to assess the safety and efficacy of TYRX in reducing CIED associated infection.^2^ The complementary use of TYRX was demonstrated to result in a significantly lower incidence of major CIED infections with or without the presence of haematoma.^3^ Based on the above, TYRX has been endorsed by Heart Rhythm Society to prevent CIED infection, especially in patients with high risk of infection.^4^

Nevertheless, there is little evidence demonstrating the degradation process of TYRX in humans. Current pocket histology studies are majorly preclinical and conducted in ovine.^5,6^ There are only two human studies characterizing the histology finding of TYRX were done on 9-year^7^ and 6.8-year^8^ ‘mature’ pocket histology. Here, we presented a case with an 8-week cardiac resynchronization therapy pacemaker (CRT-P) with TYRX received lead revision. We examine her pocket histology and compare it with the available literatures in English.

## Summary figure

**Figure:**
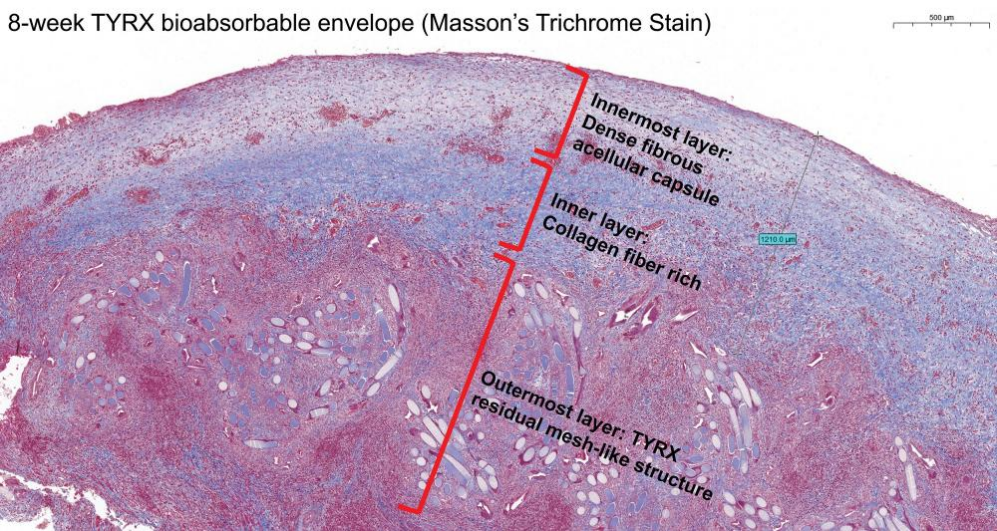


## Case

**Figure 1:**
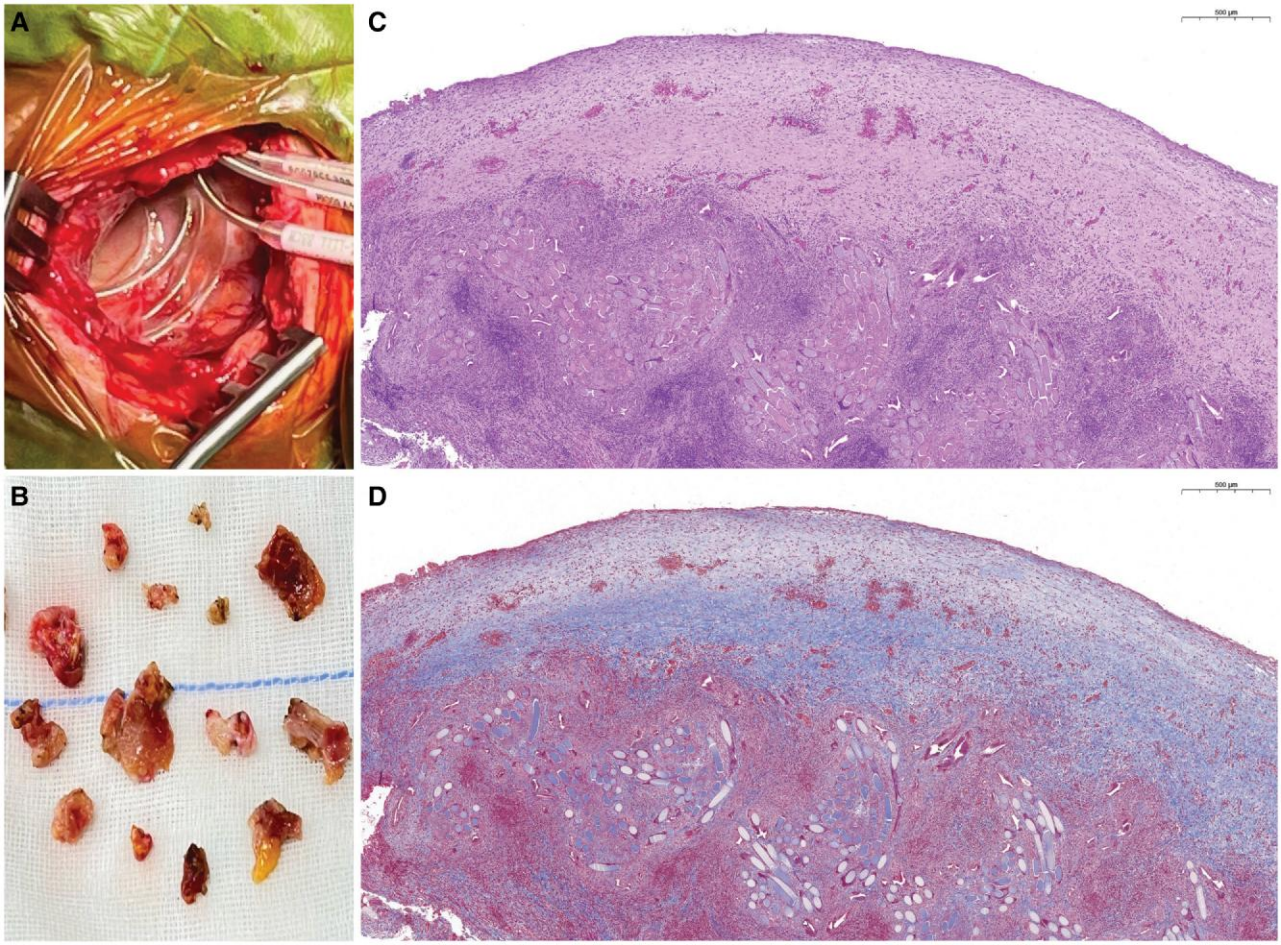
(*A*) Exposed pockets 56 days after cardiac implantable electronic device implantation with TRX envelope. (*B*) Tissue specimens of the capsule during cardiac implantable electronic device lead revision. (*C*) Cross-section of cardiac implantable electronic device pocket with haematoxylin and eosin stain. (*D*) Histopathological examination with Masson’s trichrome stain.

An 80-year-old female with hypertension, hyperlipidaemia received a CRT-P for heart failure with reduced ejection fraction and left bundle branch block. The device was wrapped with TYRX absorbable envelope to prevent pocket infection anticipated long procedure time. She remained well until one week prior to this admission (one-and-half months after the CRT-P procedure) when intermittent loss of left ventricular lead pacing developed. Pacemaker interrogation revealed low P wave amplitude (0.2 mv), which resulted in the undersensing of atrial signal, leading to failure of atrial sensing and subsequent inhibition of biventricular pacing in the atrial-tracking mode. The right atrial lead also demonstrated a high pacing threshold, suggesting possible micro-dislodgement. While the electrical parameters of both ventricular leads remained normal, she underwent a right atrial lead revision 56 days after prior CRT-P implantation. Upon opening the pocket, the generator and leads were covered with a thin capsule (*Figure 1A*). No dislodgement, broken or fracture of right atrial lead insulation. We resected the pocket and removed the fibrotic tissue. The atrial lead was replaced successfully, and the device was wrapped with a new TYRX envelope before the wound closure. The removed tissue appeared light amber and was collected for histopathology study (*Figure 1B*). The patient had a smooth postoperative hospital course and outpatient follow up.

**Figure 2:**
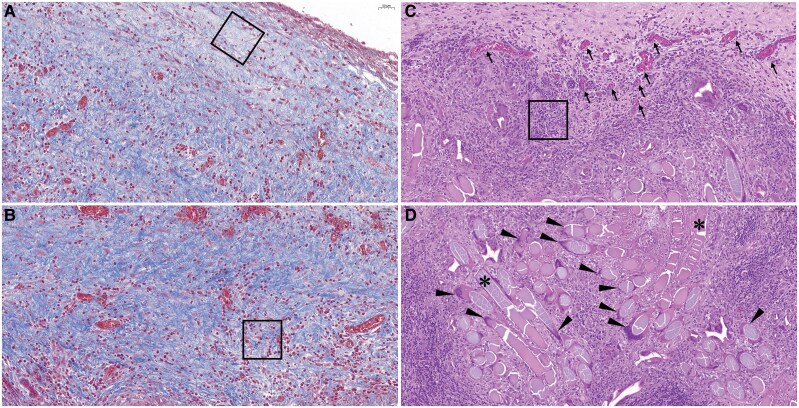
(*A*) Masson’s Trichrome stain of inner layer of the cardiac implantable electronic device pockets showed dense fibrous acellular capsule (rectangle box). (*B*) Masson’s Trichrome stain of central layer showed granulation tissue with prominent fibrosis and reduced inflammatory cells (rectangle box). (*C*) Haematoxylin and eosin stain showed lymphocytes infiltration (rectangle box) and neovascular region with Red Blood Cell in blood vessel (small arrow). (*D*) Much TYRX residual materials (asterisk symbol) are surrounded by large number of lymphocytes. Multinucleated giant cells are located near the TYRX materials (black arrowhead).

Microscopically, histopathological examination with haematoxylin and eosin (HE) and Masson’s trichrome (MT) showed a process of TYRX resorption and capsule formation (*Figure 1C* and *D*). The cross-sections of pocket tissue displayed different phases of wound healing. At the outermost layer from the CIED, prominent TYRX residual materials in mesh-like structure were surrounded by lymphocytes. Some local macrophages fused into multinucleated foreign body giant cells near the TYRX materials (*Figure 2D*). In the inner layer, neovascularization and granulation tissue consisting mainly of collagen fibres with fewer lymphocytes and macrophages replaced the envelope materials and majority of inflammatory cells (*Figure 2C* and *B*). The innermost layer, adjacent to the CIED, contained an acellular fibrous band. The collagen fibres here were densely cross-linked, retracted, and formed peridevice capsule (*Figure 2A*). The maximum thickness of capsule (the inner layer and innermost layer) measured 1210 µm.

## Discussion

Literatures have shown TYRX envelopes degrade completely around 63 days after the CIED implantation. According to limited animal studies,^5,6^ the resorption time of TYRX in an ovine model was more than 12 weeks. The wound healing process involves four main phases, including acute inflammation (polymorphonuclear neutrophils, macrophages) within 7 days, chronic inflammation (lymphocytes, multinucleated giant cells), proliferation and fibrotic (neovascularization and fibrous tissue) in the following weeks, and remodelling phases (fibrous capsule) after around 1 month.^6^  *Table 1* summarizes recent important human and animal studies. Interestingly, our histopathology study of an 8-week-old TYRX in CIED pocket displayed three phases in different layers of pocket. The outermost layer contained residual envelope materials with multinucleated foreign body giant cells, and great infiltrates of lymphocytes, which suggested a chronic inflammation phase secondary to foreign body response. Inside this layer, remarkable collagen fibres interspersed with much fewer inflammatory cells and some neovascularization. This indicated the proliferative and fibrotic stages. The innermost layer was in remodelling phase showing an acellular dense fibrous capsule with variable thickness. Of note, much TYRX material remained at this stage despite the capsule being formed. It might take weeks for the wound to achieve complete envelope residuals resorption. Thus, many lymphocytes and multinucleated giant cells were noted in the outer layer. It is not clear the impacts of TYRX degradation time, capsule formation time, and drug releasing time on the wound healing and infection prevention. More human pathology data may help the adaptation of future design of the envelope.

**Table 1:** Previously reported histology findings related to TYRX

Author	Species	Time from TYRX implantation	Finding
R. Virmani *et al*.^6^	Ovine	Day 3 Day 7 Day 28 Week 12	Mixed-type inflammatory cells predominantly polymorphonuclear neutrophils on Day 3. (HE) Interfibrillar spaces filled by granulation tissue made up of fibroblasts and macrophage with blood vessels formation on Day 7. Multinucleated giant cells were consistently encountered. (HE) Prominent fibrous tissue coat consisted mostly of Type III collagen on Day 28. Presence of multinucleated giant cells. (HE) (MT) Capsule and fibrosis histological along with maximum capsule width on Week 12. TYRX is almost completely absorbed. (MT)
G. Massaro *et al*.^7^	Human	9.0 ± 2.6 years	30 patients with mean time from the last device procedure of 9.0 ± 2.6 years. TYRX was used in 11 patients. Histological analysis of Mean capsular thickness (0.8 ± 0.3 mm in the anterior wall, 1.1 ± 0.4 mm in the posterior wall) (HE)(AMT) Neovascularization (on 8 samples from anterior wall, 13 from the posterior wall) (AMT) Inflammation (granulomatous or non-granulomatous giant cell reaction, related to implanted leads in two samples from anterior wall, and five samples from posterior) Chronic lymphocytic-macrophagic non-granulomatous inflammation on 86.7% of cases in anterior part of device pocket and 93.3% in the posterior part, equally distributed between capsular and subcapsular tissue. (HE) (immunostaining with CD3/CD20/CD68 surface marker) Calcifications (∼20% of capsules) (HE)
F. Philippon and E. Ladich *et al*.^8^	Human	6.8 ± 1.3 years	Thirty patients with existing CIED previously implanted with an absorbable TYRX. One patient had an inadequate tissue sample and two had posterior tissue samples only. Twenty-seven anterior tissue samples were analysed. Mean time from the last device procedure of 6.8 ± 1.3 years. Histological study revealed: Significantly less adhesions in the connector area and wrap area without correlation to implant duration or number of previous procedures No clinically significant calcification (Von Kossa stain) TYRX capsules contained >60% Type I collagen and were thinner (0.33 ± 0.16 mm) Nearly all (26/27) TYRX pockets had low inflammation having absent to mild inflammation
C. J. Love *et al*.^9^	Ovine	Week 12	Histopathology study of fibrous tissue capsule on Week 12 revealed minimal inflammation with healed fibrous tissue capsule. There was residual envelope material. (HE)(MT)
R. Virmani *et al*.^5^	Ovine	Week 4 Week 12	Neutrophil infiltration was found to decline to absent on Week 4 and Week 12. (HE)(MT) Increased fibrosis on Day 7 and substantial resolution by Week 12. (HE)(MT) Capsule thickness peaked at week 4

HE, haematoxylin eosin stains; MT, Masson’s trichrome stain; AMT, Azan-Mallory trichrome stain; CIED, cardiac implantable electronic device.

Our case showed the capsule thickness varied from 0.67 to 1.21 mm at 8 weeks after the procedure. Although the outer layer of capsule had not completed the remodelling, the inner portion looked well organized with adequate thickness. Massaro^7^ showed the thickness of human 9-year-fibrous CIED capsules measured 0.8 ± 0.3 mm in the anterior wall and 1.1 ± 0.4 mm in the posterior wall. Philippon *et al.*^8^ reported a mean capsule thickness of 0.33 ± 0.16 mm. In our case, we used the second-generation TYRX absorbable antibacterial envelope (Medtronic Inc., Minneapolis, MN). Previous studies have shown second-generation TYRX envelopes and next-generation TYRX envelopes both cause pocket wounds less inflammation, rapid provisional matrix formation, faster absorption, and thinner capsules.^6^ Recent publication by Philippon *et al.*^8^ reported decreased adhesions with no clinically significant calcification and low inflammation of human 6.8-year TYRX pockets. While another envelope was composed of porcine intestinal perforated, multilaminated sheets of decellularized extracellular matrix (CanGaroo®, Elutia Inc., Silver Spring, MD), exhibited increased acute and chronic inflammation and thicker capsules.^6^

In our institution, the TYRX envelope is used selectively in patients at increased risk of cardiac implantable electronic device (CIED) infection, such as those undergoing device replacement, upgrade, or lead revision, and those with chronic kidney disease, diabetes, or prolonged procedure time. Our prior real-world data showed no infections in the envelope group compared with 1.3% in controls, supporting its benefit in high-risk patients.^10^ Risk stratification is guided by the PADIT and BLISTER scores, which incorporate procedural and clinical factors to predict infection risk.^11,12^ Based on these models, extended CRT-P implantation time led to an increased risk (BLISTER score = 6, PADIT score = 4), warranting envelope use in accordance with current evidence and practice recommendations.

## Conclusion

Studies investigating human histopathology of TYRX resorption in different time points are technically difficult for ethical issues. To the best of our knowledge, we reported the first human histopathology of an 8-week-CIED pocket with TYRX. Our findings showed the capsule thickness, inflammatory patterns, envelope residuals were compatible with those in animal studies. However, this is only a case report. More reports or case series are important to explore the human pocket healing process.

## Data Availability

All available data is presented within the manuscript.

## Lead author biography

Kee Koon Ng, a practicing cardiologist at China Medical University Hospital in Taiwan, is board-certified in Internal Medicine and Cardiology.

## References

[1] Tarakji KG, Mittal S, Kennergren C, Corey R, Poole JE, Schloss E, et al Antibacterial envelope to prevent cardiac implantable device infection. N Engl J Med 2019;380:1895–1905. 30883056 10.1056/NEJMoa1901111

[2] Mittal S, Wilkoff BL, Kennergren C, Poole JE, Corey R, Bracke FA, et al The world-wide randomized antibiotic envelope infection prevention (WRAP-IT) trial: long-term follow-up. Heart Rhythm 2020;17:1115–1122. 32087357 10.1016/j.hrthm.2020.02.011

[3] Tarakji KG, Korantzopoulos P, Philippon F, Biffi M, Mittal S, Poole JE, et al Infectious consequences of hematoma from cardiac implantable electronic device procedures and the role of the antibiotic envelope: a WRAP-IT trial analysis. Heart Rhythm 2021;18:2080–2086. 34280568 10.1016/j.hrthm.2021.07.011

[4] Blomstrom-Lundqvist C, Traykov V, Erba PA, Burri H, Nielsen JC, Bongiorni MG, et al European Heart Rhythm Association (EHRA) international consensus document on how to prevent, diagnose, and treat cardiac implantable electronic device infections-endorsed by the Heart Rhythm Society (HRS), the Asia Pacific Heart Rhythm Society (APHRS), the Latin American Heart Rhythm Society (LAHRS), International Society for Cardiovascular Infectious Diseases (ISCVID) and the European Society of Clinical Microbiology and Infectious Diseases (ESCMID) in collaboration with the European Association for Cardio-Thoracic Surgery (EACTS). Europace 2020;22:515–549. 31702000 10.1093/europace/euz246 PMC7132545

[5] Virmani R, Kassotis J, Mittal S, Philippon F, Kudlik D, Kirchhof N, et al Effects of envelopes on CIED pocket healing: a head-to-head preclinical evaluation. Eur Heart J 2022;43:ehac544.741. 10.1016/j.hrthm.2024.02.054 38555971

[6] Virmani R, Philippon F, Mittal S, Finn A, Kudlik DA, Kirchhof N, et al Effects of envelopes on CIED pocket healing: a head-to-head pre-clinical evaluation. Heart Rhythm 2024;21:1325–1333. 38555971 10.1016/j.hrthm.2024.02.054

[7] Massaro G, Leone O, Valzania C, Angeletti A, Corti B, Martignani C, et al Pocket histology at cardiac implantable electronic device replacement: what's new? Heart Rhythm 2023;20:198–206. 36309157 10.1016/j.hrthm.2022.10.017

[8] Philippon F, Ladich E, Virmani R, Ip JE, Wright J, Andrew Hazlitt H, et al An absorbable antibacterial envelope promotes development of a healthy device pocket: results of the TYRX pocket health study. J Cardiovasc Electrophysiol 2025;36:1978–1986. 40521692 10.1111/jce.16766

[9] Love CJ, Hanna I, Thomas G, Greenspon AJ, Christie M, Goodman J, et al Preclinical evaluation of a third-generation absorbable antibacterial envelope. Heart Rhythm 2023;20:737–743. 36693614 10.1016/j.hrthm.2023.01.018

[10] Chang CF, Tang WD, Chen YH, Lu CR, Chung WH, Lin CL, et al Antibacterial envelope prevents cardiac implantable electronic device infections: the largest Asia real world data. Acta Cardiol Sin 2025;41:314–322. 40416565 10.6515/ACS.202505_41(3).20250107A PMC12099244

[11] Ahmed FZ, Blomstrom-Lundqvist C, Bloom H, Cooper C, Ellis C, Goette A, et al Use of healthcare claims to validate the Prevention of Arrhythmia Device Infection Trial cardiac implantable electronic device infection risk score. Europace 2021;23:1446–1455. 33755136 10.1093/europace/euab028 PMC8427456

[12] Maclean E, Mahtani K, Honarbakhsh S, Butcher C, Ahluwalia N, Dennis ASC, et al The BLISTER score: a novel, externally validated tool for predicting cardiac implantable electronic device infections, and its cost-utility implications for antimicrobial envelope use. Circ Arrhythm Electrophysiol 2024;17:e012446. 38258308 10.1161/CIRCEP.123.012446 PMC10949977

